# Dealing With Conflicts in Medical Decisions: Epistemic Reasonable Disagreement Between Parents and Medical Staff

**DOI:** 10.1111/bioe.70125

**Published:** 2026-05-29

**Authors:** Chiara Innorta

**Affiliations:** ^1^ University of Eastern Piedmont, FINO Convention Vercelli Piedmont Italy

**Keywords:** communication, conflicts between parents and medical staff, emotions, epistemic injustices, shared decision‐making, the Gard case

## Abstract

Many controversies in medical ethics, particularly those involving conflicts between parents and medical staff over decisions about child patients, are challenging to manage without causing significant polarization and communication issues. This is primarily because the parties involved—parents and physicians—operate at different epistemic levels, engaging with and relying on distinct forms of knowledge. A key issue underlying this epistemic disparity—the imbalance in how each party's knowledge is recognized and valued—is the difficulty of achieving what I term epistemic reasonable disagreement. This concept is crucial for fostering a shared decision‐making process in which parents and physicians recognize each other's equal epistemic value, acknowledging that both perspectives are equally credible and understandable. Establishing full credibility for the parental position can be problematic because it is typically grounded in emotions, a quality often undervalued in bioethics. This paper argues, however, that the tendency to dismiss emotions can be mitigated by applying the principle of charity. Achieving reasonable epistemic equality allows for the recognition of parents' epistemic contributions as a form of knowledge. Applying a framework for epistemic reasonable disagreement can prevent testimonial and hermeneutical injustice towards parents and encourage a re‐evaluation of how dialogue between parents and physicians should be approached.

## 1 Introduction

This study examines disagreements between parents and physicians over end‐of‐life decisions for children, applying the concept of epistemic reasonable disagreement to understand how these conflicts emerge and are managed through communication between both parties. Rather than arguing that recognizing parents' viewpoints should lead to different clinical decisions (e.g., agreeing to parents' requests), the focus here is on how such recognition can foster a more empathic, constructive process of communication and shared decision‐making by defusing confrontation, supporting mutual understanding, and encouraging greater patience with families.

The Gard case [1, 2, 3] is a paradigmatic example in which the conflict between parents and physicians displayed communication and trust issues.^1^ Born in 2016, Charlie Gard was diagnosed with infantile‐onset mitochondrial DNA depletion syndrome (RRM2B mutation), a rare genetic disorder with a poor prognosis. Although his parents and Great Ormond Street Hospital (GOSH) initially agreed to pursue experimental nucleoside therapy, clinicians eventually deemed the treatment futile and proposed withdrawal of life‐sustaining treatment, which started to put the different perspectives in conflict. When the parents fundraised to seek treatment in the United States, GOSH sought legal intervention, likely weakening mutual trust. Court rulings consistently favored GOSH amid intense media scrutiny. The parents ultimately accepted the court's decision in July 2017.

An editorial of *The Lancet* in the aftermath of the case acknowledged that, together with the medical and legal discussion, “at the heart of this sad story is a breakdown of trust and communication between Charlie's parents and the clinical team at GOSH” [8]. Judge Francis himself, in his final statement, reinforces the value of analyzing the Gard case through the lens of failed communication: “However, it is my clear view that mediation should be attempted in all cases such as this one even if all that it does is achieve a greater understanding by the parties of each other's positions” [9, p. 6]. Is the conflict in cases like this aggravated by epistemic injustice? Could the conflict have been reduced by applying a framework of epistemic reasonable disagreement?

These problems in trust and communication arise partly from the different sources of knowledge underlying medical expertise and parental testimony. Physicians' expertise inherently grounds the credibility of their views,^2^ while their own emotional reactions frequently remain unacknowledged, even though they play a significant role in patient care [11, 12]. ^3^ On the other hand, the parental perspective often does not receive full recognition as credible and therefore is not satisfactorily understood, largely because it is grounded in emotions which are partially undervalued in the medical context [14, 15].

In this scenario, two epistemic subjects, both capable of contributing knowledge, are positioned at different levels, which can harm dialogue from the outset. However, this situation could be solved by achieving a condition of epistemic reasonable disagreement, which requires treating both parties as possessing relevant knowledge and understanding within the shared decision‐making process.^4^ Shared decision‐making is widely supported in healthcare policy as a way to promote such epistemic equality, but in practice it often fails to achieve equal participation: patients are frequently treated as passive, excluded from model development, and face barriers such as limited knowledge, power imbalances, and a lack of confidence, while clinicians cite time constraints and doubts about patient engagement [19, 20].

To address these challenges and develop the framework of epistemic reasonable disagreement, the paper is structured as follows. Section 2 provides an overview of epistemic injustice in healthcare, with a specific focus on the parental role in cases of ill children. Section 3 explains the theoretical foundations of the proposal, rooted in Rawls's reasonable disagreement concept, as well as in Rorty's and Nussbaum's approaches, which are used to justify the reasonableness of parental emotions. Section 4 presents the core concept of epistemic reasonable disagreement, contrasting it with Wilkinson and Savulescu's interpretation of reasonable disagreement. Section 5 explores the practical implications of this concept, introducing the idea of “reasonable epistemic equality” and its potential application for Clinical Ethics Support (CES). Section 6 acknowledges the potential limitations of the approach and Section 7 concludes.

## 2 An Epistemic View of the Healthcare Setting

Carel and Kidd studied the healthcare context through an epistemic lens, highlighting a widespread communication problem between patients and physicians [21]. This creates a challenging dynamic in which both sides struggle to understand one another. Some critics link these epistemic concerns to a broader crisis in modern medicine and the need for a more humanistic approach [22]. In line with these critiques, Ofri's examples further illustrate how marginalizing emotions in medical practice can create epistemic barriers between patients and physicians [23].

### 2.1 Testimonial and Hermeneutical Injustice

Carel and Kidd suggest that ill persons can be victims of testimonial injustice, as they are often labelled with negative characteristics, such as cognitive unreliability and emotional instability [24, 25]. According to Fricker, “testimonial injustice occurs when prejudice causes a hearer to give a deflated level of credibility to a speaker's word” [26, p. 1].

For ill persons, many aspects of the experience of illness are difficult to understand and to communicate. This is the result of what can be called hermeneutical injustice, which “occurs at a prior stage, when a gap in collective interpretive resources puts someone at an unfair disadvantage when it comes to making sense of their social experiences” [26, p. 1].

Medical experts tend to dismiss the relevance of information expressed by patients [27]. The reason for this tendency is twofold. First, patients are often perceived as vulnerable individuals whose irrationality is thought to overshadow their reasoning ability [28]. Second, ill persons are often unable to use the accepted language of medical discourse. It is as if physicians and patients experience illness from within different “worlds” [29]. This inherent difference makes it more challenging for physicians to cultivate empathetic understanding when patients are increasingly perceived as dissimilar.

### 2.2 Testimonial and Hermeneutical Injustices Towards Parents

Testimonial and hermeneutical injustices also apply to parents in the cases we are considering. Parents are frequently represented as inexperienced, emotionally overwhelmed, and having difficulty understanding key information [30]. Healthcare professionals are often expected to educate and guide parents, who are commonly portrayed as vulnerable and unable to make informed decisions [31].

However, I suggest that the testimonial injustice faced by parents is not merely an injustice in which someone is wronged specifically in their capacity as a knower, but rather an injustice in which parents are wronged in their capacity as knowers of their own reasons. Although parents may not provide purely factual “testimony” in the clinical sense, their intimate knowledge and interpretations of their child's experiences are indispensable for fostering stable and collaborative communication. Therefore, it is crucial to understand and give credibility to their reasons, which establish their epistemic role as knowers.

At the same time, parents can experience hermeneutical injustice, as the parental group may suffer from a gap in collective resources that places them at an unfair disadvantage in communication. In particular, this form of injustice arises because the resources needed to interpret parents' socially embedded experiences—largely grounded in the emotional component—are not fully acknowledged within the dominant hermeneutical framework. Incorporating emotions enables bioethics to grasp more deeply how feelings shape shared decision‐making [32].

## 3 Theoretical Foundations of Epistemic Reasonable Disagreement

### 3.1 Rawls's Framework

The concept of reasonable disagreement derives from Rawls's political philosophy. Rawls defines reasonable disagreement as a “disagreement between reasonable persons: that is, between persons who have realized their two moral powers to a degree sufficient to be free and equal citizens in a constitutional regime, and who have an enduring desire to honor fair terms of cooperation and to be fully cooperating members of society” [33, p. 55]. Thus, citizens are free to exercise their freedom regarding moral choices and develop their comprehensive doctrines.

There can be difficulties in defining reasonableness in Rawls's work.^5^ Nevertheless, there is consensus that reasonable disagreement enables a just and stable society of free and equal citizens to endure over time [35]. To be reasonable, then, a citizen must develop a specific moral attitude in order to listen to the reasons of others. Reasonableness is thus understood as the ability to tolerate others who hold reasonable yet conflicting comprehensive moral doctrines. In Rawls's work, it is primarily a political and moral ideal of democratic citizenship.

The concept of reasonable disagreement in the healthcare context cannot be directly applied in the moral sense proposed by Rawls. I contend that the perspectives of parents and medical staff should not be viewed as two distinct comprehensive moral doctrines, but rather as stemming from different epistemic sources. Parents' perspectives are grounded in experiential knowledge and values, shaped by emotions that influence their decision‐making. By contrast, the physicians' viewpoint—while also rooted in values and shaped by emotions—is often guided primarily by empirical evidence and professional medical guidelines.

Nonetheless, the priority Rawls places on the capacity to tolerate and understand positions different from our own is key in the proposed approach. Indeed, the communication gap between parents and medical staff could be reduced by developing new ways to interpret parents' emotion‐based beliefs more charitably, in a setting where both sides are equally listened to and respected—that is, on an epistemically level playing field.

### 3.2 The Reasonableness of Parental Emotions

To address the epistemic injustices affecting parents, it is crucial to justify the reasonableness of parental emotions. I will build on the principle of charity applied to communication, alongside Rorty's and Nussbaum's analyses of emotions.

#### 3.2.1 Rorty's Approach

The principle of charity requires that a person's beliefs be interpreted as plausibly as possible [36]. It also demands a shared sense of reasonableness, a crucial concept for social understanding [37]. In the healthcare context, this would require physicians to adopt an interpretative approach to medical practice, modelled on dialogue‐based hermeneutics [38]. Physicians, along with other healthcare professionals, act primarily as interpreters of health and illness [39]. Dialogical hermeneutics requires a context characterized by mutual openness, in which participants remain open to revisiting and reshaping their interpretations. This dialogical space is not given in advance but is co‐created through interaction, sustained by trust, shared generosity, compassion, and authentic engagement among participants [40].

In the dialogue between parents and physicians, Rorty's proposal of using the principle of charity to explain the origin of an emotion [41] becomes central. Here, the principle of charity serves as an interpretative guideline that encourages the adoption of the most favorable interpretation when describing someone's emotions. This means approaching another person's emotions or words according to what Gadamer calls a “principle of perfectness”: we should interpret the other in the best light, presuming sense and truth rather than error, so that dialogue can generate new understanding instead of remaining at the level of dismissal or misunderstanding [38].

Rorty's application of the principle of charity is valuable both for reconstructing a person's intentional system and for establishing the rationality of someone who holds a justified emotion: “It is not the belief or emotion that is rationalized, but a person's coming to have it” [41, p. 141]. This implies, in my view, that the parental position qualifies as reasonable, even if the emotions or beliefs involved remain open to critical examination. Parents' emotions emerge from deeply ingrained values, personal experiences, and cultural narratives about parental duty and care. From Rorty's standpoint, to assess the reasonableness of these emotions, one must examine their genesis and underlying reasons, rather than merely judging their outward expression.

Applying the principle of charity provides then the foundation for the parents' viewpoint, granting them epistemic credibility and preventing the occurrence of epistemic injustices. Genuine understanding involves moving beyond individual perspectives to achieve a fusion of horizons, fostering a new shared understanding that incorporates elements of each party's original viewpoint [38, pp. 301–305].

Nordby, for example, shows how nurse‐patient interactions in medical settings can be guided by the principle of charity [42], a view supported by Rorty, who advocates engaging with emotions through dialogue as opportunities for deeper understanding rather than as obstacles to reason.

#### 3.2.2 Nussbaum's Approach

Nussbaum's perspective on human emotions provides another related foundation for understanding the reasonableness of parental emotions [43]. According to Nussbaum, emotions are closely tied to cognitive evaluations. These evaluations are profoundly shaped by societal norms, cultural expectations, and individual experiences. As societal values evolve, so too do the emotional responses of individuals within that society [44].

Furthermore, Nussbaum underscores that an individual's emotional life is inseparable from the “emotional grammar” of their society. This means that those who do not share a cultural framework may not experience emotions in the same way as those who do [43]. This insight is particularly relevant in the context of parental emotions in medical decision‐making.

Parental emotions need to be interpreted within the social and cultural frameworks that shape them. Ignoring these roots risks unfairly dismissing parents' perspectives, whereas acknowledging them can reveal their reasonableness.

## 4 Developing the Concept of Epistemic Reasonable Disagreement

### 4.1 Savulescu and Wilkinson's Concept of Reasonable Disagreement

Savulescu and Wilkinson introduced the concept of reasonable disagreement in relation to the medical‐ethical controversies arising in the Gard case [2]. In their analysis of the Gard case, they suggest that the concept of reasonable disagreement may be retrospectively applicable, provided that a condition of reasonable dissensus among the physicians can be identified. Reasonable dissensus refers to the view that, in cases involving treatment limitation, discussions should be held with families, and decisions may proceed if at least one member of the medical team supports the proposed course of action after thorough reflection [45].

Although they argue that healthcare professionals and parents bring diverse values to decision‐making [2, p. 106), their analysis does not adequately recognize the credibility of the parental perspective. I argue that their account concentrates mainly on disagreements within the medical team itself, rather than on the dialogue between professionals and patients. While patient values may be voiced by clinicians, they are often only indirectly represented. The approach proposed here aims to foster direct dialogue between patients and professionals, articulating the necessary epistemic conditions for achieving genuine equality of testimony in approaches such as moral case deliberation (MCD) [46]. Through this dialectical process, the exchange becomes a learning environment where differences are explored. However, in groups of professionals and patients, tensions can emerge from differences in roles, interests, language, and communication styles.

### 4.2 Towards Epistemic Reasonable Disagreement

The concept of epistemic reasonable disagreement, inspired by Rawls's concept of reasonable disagreement, can be defined in this way: “Epistemic reasonable disagreement is a condition where patients and physicians, by mutually acknowledging each other's epistemic power and thus crediting their distinct forms of knowledge, commit to respectfully accepting differing perspectives to foster cooperative exchange and dialogue.”

The framework proposed in this paper is inspired by the epistemology of disagreement, which explores the epistemically relevant aspects of interactions between disagreeing parties [16, 18, 47, 48, 49]. A key challenge within this field, which is particularly relevant in our context, arises in situations where disagreeing parties are uncertain whether their interlocutor genuinely qualifies as an epistemic peer. This uncertainty often stems from insufficient knowledge of the other person's background or the controversial nature of the topic, making it difficult to judge their epistemic standing accurately. In healthcare, this ambiguity often defaults to a system where physicians are assumed to hold exclusive epistemic authority [50]. Recognizing that this initial uncertainty in the medical context has traditionally led to the subordination of the parental role is the first step towards identifying the problem and moving towards the application of epistemic reasonable disagreement.

Moreover, it is only possible to achieve a condition of epistemic reasonable disagreement if the parties involved are able to attribute epistemic credibility to one another—that is, if each side sees the other's knowledge and viewpoint as reliable and worthwhile, and believes that the other can make a useful contribution to the discussion. By “credibility”, I refer to the epistemic tradition in which a subject's credibility depends on recognition by the listener, without undermining the subject's capacity as a knower [26]. Therefore, when individuals are considered credible, it is entirely justifiable and understandable that they express their viewpoints.^6^

Thus, the concept of epistemic reasonable disagreement requires that:

− The subjects involved in the disagreement must be recognized as fully credible and feel genuinely understood, so that their perspectives, underlying reasons, and the full weight of their contributions are accurately grasped and meaningfully acknowledged by the other party.
− The subjects involved in the disagreement must behave as epistemic peers, approaching the disagreement with mutual intellectual respect and openness to learning by engaging with each other's reasons and evidence, rather than simply deferring to or dismissing the other based on status.

It is crucial to distinguish the concept of epistemic reasonable disagreement from epistemic relativism [54]. This framework is not aimed at determining who is right or wrong, nor does it treat competing claims as equally true. Instead, it seeks to create an epistemic space for communication where different forms of knowledge are given equal consideration and respect. However, an epistemic “unreasonable” disagreement arises when parents demand actions that endanger the child or contradict established medical knowledge (e.g., refusing a life‐saving transfusion or insisting on treatment in cases of brain death). Conversely, unreasonable positions can also originate from physicians. Indeed, clinical decisions can be influenced by physicians' personal or controversial standpoints, sometimes diverging from what is supported by rigorous clinical evidence [55]. For instance, a neonatologist with a sanctity‐of‐life perspective may push for aggressive resuscitation of a 21‐week infant, downplaying clinical evidence [56]. In such cases, parents may agree with the physician, resulting in an “unreasonable agreement”. These situations demonstrate that simply recognizing parental and medical positions has significant limits in overcoming communication barriers when those perspectives are inherently unreasonable.

However, I view the framework of epistemic reasonable disagreement—grounded in epistemic justice and communicative rights—as a necessary condition to implement the type of shared decision‐making process proposed by Galasiński et al. [57]. This framework would enable disagreements to be managed in practice with trust, tolerance and reciprocity [58].

## 5 Epistemic Equality and Parental Knowledge: Practical Consequences

Power imbalances can hinder the creation of shared knowledge, but ensuring epistemic equality in the processes of reasoning and justification helps to overcome these barriers [59]. In this paper, I apply a model of epistemic equality to the debates between parents and medical staff, which I refer to as reasonable epistemic equality. Reasonable epistemic equality can be defined as follows: “Reasonable epistemic equality is a practical state in which physicians and parents, as epistemic subjects, equally participate in shared decision‐making, where this equality is considered reasonable because neither viewpoint is more reasonable than the other.” For clarity, epistemic reasonable disagreement describes the theoretical framework that justifies physicians and parents as credible epistemic peers. Reasonable epistemic equality, in turn, represents the effective realization of this framework in concrete exchanges, where the reasonableness of each viewpoint is given equal consideration.

Achieving reasonable epistemic equality allows for the recognition of parents' epistemic contributions, namely the valuable insights, observations, and experiences that genuinely enhance the collective understanding of the child's condition. This aligns with Fricker's concept of “epistemic contribution”, which she recognizes as a fundamental social epistemic capability [60]. She defines this capability as fostering an individual's ability to contribute to and engage with shared knowledge resources, enhancing collective understanding and supporting informed, cooperative decision‐making within a community. This is a core concept for understanding epistemic relational equality and inequality.^7^ In the medical context, it highlights that each party's capacity to function as an epistemic contributor is inherently relational, depending crucially on supportive connections and a communal environment.

A dialogue characterized by reasonable epistemic equality can be understood as a knowledge‐gathering phase in which all perspectives are shared openly, creating an epistemic space where both parties work to connect diverse perspectives.

### 5.1 Rethinking the Gard Case and Other Similar Cases

If communication and management in the Gard case had fully embodied the principles of epistemic reasonable disagreement—using strategies to reduce conflict, build respect, and manage disagreements constructively—this approach could have fostered trust and transparency [61], ensuring that all parties were granted epistemic credibility, even if agreement was not guaranteed.

For instance, in the Gard case, the physicians' initial willingness to pursue experimental treatment suggests a recognition of the parents' perspective. However, as medical opinions shifted, it is likely that the decision‐making process became more adversarial. A more charitable approach to parental beliefs might have acknowledged that their insistence on treatment was based on deeply held emotions that had a rational and societally shaped origin.

To address such shifts and support collaborative decision‐making, establishing a shared knowledge base, guided by reasonable epistemic equality, could have strengthened the process from the outset. This process would involve holding an initial meeting to collaboratively map all participants' knowledge, beliefs, values, experiences, and uncertainties, potentially using a whiteboard.^8^ The whiteboard methodology enhances communication between patients and providers. It also facilitates patient engagement with their care by clearly identifying team members and improving understanding of care goals [63].

To foster openness and understanding, clinical staff can be trained in structured communication techniques, including narrative interviewing, which encourages individuals to share significant life experiences from their unique perspectives [64]. Narrative interviews prioritize the storyteller's perspective by avoiding the imposition of a predefined agenda [65]. This approach can be particularly useful for revealing the reasonableness of the origin of the emotions expressed in parental discourse.

The epistemic reasonable disagreement approach directly influences how Clinical Ethics Support (CES) is organized and evaluated, thereby helping healthcare staff to navigate ethical challenges [66]. Its goal is to create a physical and intellectual space that enables deep, experiential communication between physicians and parents. It emphasizes recognition of the epistemic value of all parties involved—a recognition that has direct implications for both the moral dimension and the nature of the dialogue itself. Strengthening epistemic credibility can improve how moral conflicts over difficult medical decisions are understood. Much of the feasibility of this approach depends on physicians who, through practice and education, are expected to develop a more empathetic and robust emotional engagement with patients. The proposed approach aligns with other models that emphasize the value of the narrative dimension and a hermeneutic dialogical approach and advocate for a deep integration of theoretical foundations and practical experience [67, 68]. Within these frameworks, which focus on patients' concerns and emotional dimensions, theory functions not as a source of final answers but as a tool that clarifies values, supports ethical reflection, and makes complex situations more intelligible.

## 6 Limitations and Objections to This Approach

The cited cases (e.g., Charlie Gard) are used solely as illustrative examples of potential communication problems in complex shared decision‐making. Indeed, evidence also shows that physicians often make a considerable effort to understand parents' perspectives. Public accounts are necessarily partial and clinicians are bound by confidentiality; therefore, the full dynamics of these cases cannot be verified.^9^ This represents an empirical limitation of the proposed approach.

In fact, in several high‐profile UK court cases cited, there are strong indications that clinicians invested considerable effort in listening to parents, endeavoured to respect their perspectives, and likely sought to grant them epistemic credibility. These extended periods of engagement often preceded judicial involvement and were intended to facilitate agreement. Still, for various reasons, some parents ultimately do not accept the medically recommended course of action. This persistence of disagreement, even if epistemic credibility was indeed present in those interactions (which we can only assume), could be seen as another possible limitation of the approach. However, acknowledging the reasonableness of an opposing viewpoint does not necessarily lead to full and permanent agreement. The dialogical approach presented here should be regarded rather as a means to reduce conflict by fostering mutual respect and understanding even when consensus remains incomplete.

Another objection can be drawn from the Gard case, as the parents' decision to rely on independent internet research to identify alternative treatments may be criticized. This could reflect a breakdown in epistemic trust [69], in which confidence in the other party's reliability as a source of knowledge is compromised, thereby undermining communication. It could be argued that the parents placed more trust in information found online than in the advice of the physicians caring for their child on a daily basis. While much attention is given in this paper to the way parents are undervalued—framing them as victims of epistemic injustice—the reverse dynamic also merits scrutiny. Medical professionals may justifiably feel that their epistemic credibility is being unfairly challenged or ignored and may experience a form of testimonial injustice when their expertise is dismissed without adequate consideration. This objection requires further exploration to fully understand its problematic implications.

## 7 Conclusion

This paper proposes an epistemic understanding of reasonable disagreement in medical conflicts, specifically between parents and medical staff. This approach offers a theoretical basis for recognizing the validity of opposing views, thereby making genuine dialogue possible. Crucially, the epistemic dimension of reasonable disagreement emphasizes the need to recognize the contributions of both parents and medical staff as valid forms of knowledge, regardless of their origin. By using the concept of epistemic reasonable disagreement to validate the parental perspective, which is often grounded in emotion, this study explores the potential epistemic injustices experienced by parents. It suggests that parents may face difficulties not only in being listened to by physicians but also in effectively explaining and justifying their viewpoints.

A comprehensive understanding of this phenomenon requires further empirical investigation, given the inherent difficulty in obtaining complete information from specific cases to construct a detailed account. However, drawing on controversial cases such as the Gard case allows for the identification of tools for improving communication between parents and medical staff.

The paper introduces the concept of reasonable epistemic equality, defined as a space in which the knowledge of both parties is effectively shared. It is proposed that this sharing could be facilitated through methods such as the use of whiteboards and narrative interviewing, which would serve as a practical means of integrating diverse perspectives, building trust, and understanding the origin of the other party's point of view. Achieving a satisfactory level of communication involves a key prerequisite: the adoption of a charitable approach by physicians. While physicians already invest considerable effort in patient care and communication, they could nevertheless further cultivate an attitude that attributes validity to, and genuinely listens to, parental perspectives. This enhanced approach would create new perspectives and, in turn, help to more effectively reduce and constructively manage conflict.

## Conflicts of Interest

The author declares no conflicts of interest.

## Data Availability

The author has nothing to report.

## Endnotes

1 Several other high‐profile cases, such as Archie Battersbee, Alfie Evans, Isaiah Haastrup, and more recently Indi Gregory, have highlighted the ethical and legal complexities surrounding end‐of‐life decisions for critically ill children. As in the Charlie Gard case, these cases often involve conflicts between parents and medical professionals over the continuation or withdrawal of life‐sustaining treatment [4, 5, 6, 7].

2 The culture of medicine has increasingly shifted towards prioritizing rationality over emotion [10].

3 In critical care, professionals are intimately familiar with parents’ emotional responses to severe illness. It is argued that these professionals do not necessarily dismiss such emotions as invalid; rather, they typically recognize and respect them in practice. However, the depth of their understanding of the origin or full implications of these emotions for parents may not always be complete [13].

4 Two primary epistemic factors influence the epistemic standing of the parties involved in a disagreement: the quantity of evidence available to them and their cognitive resources [16, 17, 18].

5 It is useful to highlight that the concept of “reasonable” remains unclear in political debate [34].

6 It can be suggested that individuals have a fundamental need to feel acknowledged and understood [51, 52]. This sense of being understood is commonly referred to as perceived understanding [53].

7 “No matter the cultural context, the question of who gets to contribute epistemically to shared knowledge and/or shared social understandings that may be sought, canvassed, pooled, or otherwise genuinely engaged with in any given practical context is a locus of what we might usefully conceive of as epistemic relational equality and inequality” [60, p. 6]

8 However, this tool needs to be improved and verified in the proposed context [62].

9 The topic of epistemic injustice in healthcare—particularly regarding parents in paediatric conflicts—remains only partially explored. While empirical examples suggest potential challenges, we currently lack direct and unambiguous evidence to assess the epistemic dynamics with certainty. This limitation should be kept in mind when considering the hypothetical application of the proposed approach, which, nonetheless, is grounded in a solid theoretical foundation.
